# A study on the energy and exergy of Ohmic heating (OH) process of sour orange juice using an artificial neural network (ANN) and response surface methodology (RSM)

**DOI:** 10.1002/fsn3.1741

**Published:** 2020-07-11

**Authors:** Mohammad Vahedi Torshizi, Mohsen Azadbakht, Mahdi Kashaninejad

**Affiliations:** ^1^ Department of Bio‐System Mechanical Engineering Gorgan University of Agricultural Sciences and Natural Resources Gorgan Iran; ^2^ Department of Food Science and Technology Gorgan University of Agricultural Sciences and Natural Resources Gorgan Iran

**Keywords:** artificial neural network, modeling, Ohmic heating, response surface method, sour orange, thermodynamic analysis

## Abstract

The nonmodern statistical methods are often unusable for modeling complex and nonlinear calculations. Therefore, the present research modeled and investigated the energy and exergy of the ohmic heating process using an artificial neural network and response surface method (RSM). The radial basis function (RBF) and the multi‐layer perceptron (MLP) networks were used for modeling using sigmoid, linear, and hyperbolic tangent activation functions. The input consisted of voltage gradient; weight loss percentage, duration ohmic, Input flow, Power consumption, electrical conductivity and system performance coefficient and the output included the energy efficiency, exergy efficiency, exergy loss, and improvement potential. The response surface method was also used to predict the data. According to the result, the best prediction amount for energy and exergy efficiencies, exergy loss and improvement potential were in RBF network by sigmoid activation function and after this network, RSM had the best amount for energy efficiency, Also for exergy efficiencies, exergy loss and improvement potential obtained acceptable results in MLP network by a linear activation function. The worst amount was at MLP network by tangent hyperbolic. In general, the neural network can have more ability than the response surface method.

## Graphical abstract

The nonmodern statistical methods are often unusable for modeling complex and nonlinear calculations. Therefore, the present research modeled and investigated the energy and exergy of the ohmic heating process using an artificial neural network and response surface method. In general, the neural network can have more ability than the response surface method.

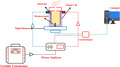

## 1 INTRODUCTION

Nonmodern statistical methods are often unusable for modeling complex and nonlinear computations, especially if there are not clear relationships between output and measured characteristics of model. The intelligent predictive methods such as artificial neural networks are computation methods that lack problems in nonmodern methods. These types of systems have properties such as learning capability, generalizability, information dispersion, parallel processing and robustness, use in pattern separation, grading, function approximation, and correlation equation, and they are generally used where there is a need to learn a linear or nonlinear mapping. Artificial neural network plays an important role in predicting process parameters as a powerful tool (Johnsson, 2018; Van Dam, 2014). On the other hand, artificial neural network is widely used in many fields. Furthermore, its use is significantly important for researchers in solving complex and nonlinear equations in dryers and energy‐consuming systems in recent years due to the fact that it uses the empirical data (Azadbakht, Torshizi, Noshad, & Rokhbin, 2018; Özdemir, Aktaş, Şevik, & Khanlari, 2017). According to studied predictive and diagnostic techniques, most artificial neural networks (ANNs) have three layers including the input, hidden, and output layers. The output result depends on the applied weight in data of connection of output and hidden layers. During the training and learning of a network, weights indicate the worth of an ANN to produce a very close result to real output (Balogun, Salami, Aibinu, Mustafah, & Sadiku Isiaka, 2014). Artificial neural networks (ANNs) are powerful modeling techniques that, in short, work with arrays of neurons in memory and biological learning. ANNs offer many advantages over conventional modeling techniques because they can provide models based on no hypothesis about the nature of phenomenological mechanisms and understanding the mathematical fields for the main problem of process, and the ability to learn linear and nonlinear relationships between variables directly from a set of samples (Fathi, Mohebbi, & Razavi, 2011). In recent decades, most researchers in agricultural engineering have used conventional learning algorithms such as artificial neural networks (Pan et al., 2016). Unlike mathematical models, neural network models are able to identify relationships between parameters without the need to extract their relationships, and thus they are considered as very powerful tools in modeling. In this method, relationships of parameters are introduced at the stage of network training, and it then acts similar to the human brain. In the case of a proper training, the neural network will be able to predict the process and solve problems of extracting relationships of parameters. Therefore, neural network models are often used in cases in which relationships of parameters are unknown or very complex (Ghasemi, Aghayari, & Maddah, 2017). Various researchers have predicted and modeled processes using the artificial neural network: Nikbakht, Motevali, and Minaei (2014) used the neural networks to predict the exergy and energy of pomegranate drying in a thin layer dryer by microwave (Nikbakht et al., 2014). Aghbashlo, Mobli, Rafiee, and Madadlou (2012) carried out experiments by an artificial neural network to predict the performance of Spry dryer in oil and fish (Aghbashlo et al., 2012). Najafi, Faizollahzadeh Ardabili, Mosavi, Shamshirband, and Rabczuk (2018) investigated an intelligent artificial neural network‐response surface methodology method for exergy and energy analysis that reported based on the mentioned results it can be concluded that employing ANN model in predicting or controlling circuit, will provide more possibility of useful work compared with that obtained by mathematical modeling (Najafi et al., 2018). Ardabili, Najafi, Ghaebi, Shamshirband, and Mostafaeipour (2017) had done a novel enhanced exergy method in analyzing HVAC system using soft computing approaches: A case study on mushroom growing hall that obtained MLP method had a poor performance in this study by linearity of 0.9511 and RMSE of 0.5584 with deviation of 12.5254 kJ/s (Ardabili et al., 2017).

The present paper aimed to investigate and predict the experimental data of the Ohmic heating method using neural networks, so that we could select a suitable network with high accuracy and speed and compare whether predicted values in the response surface method were better or predicted values by an artificial neural network.

## 2 MATERIALS AND METHODS

### 2.1 Sample preparation

The sour oranges were purchased from a garden located in the city of Gorgan, Golestan Province. The prepared oranges were washed and divided into two halves in the middle and immediately after purchase, all the samples juice was taken manually in the same conditions, and the samples were prepared to conduct the test during the Ohmic process with voltage gradients and the percentages of different weight loss to investigate the amount of energy efficiency, exergy efficiency, exergy loss, and improvement potential during the process. This experiment was done in 2019 at Gorgan University of Agricultural Sciences and Natural Resources; environmental conditions for testing were conducted at a temperature of 19°C and a relative humidity of 65%.

### 2.2 The experiment method

**FIGURE 1:**
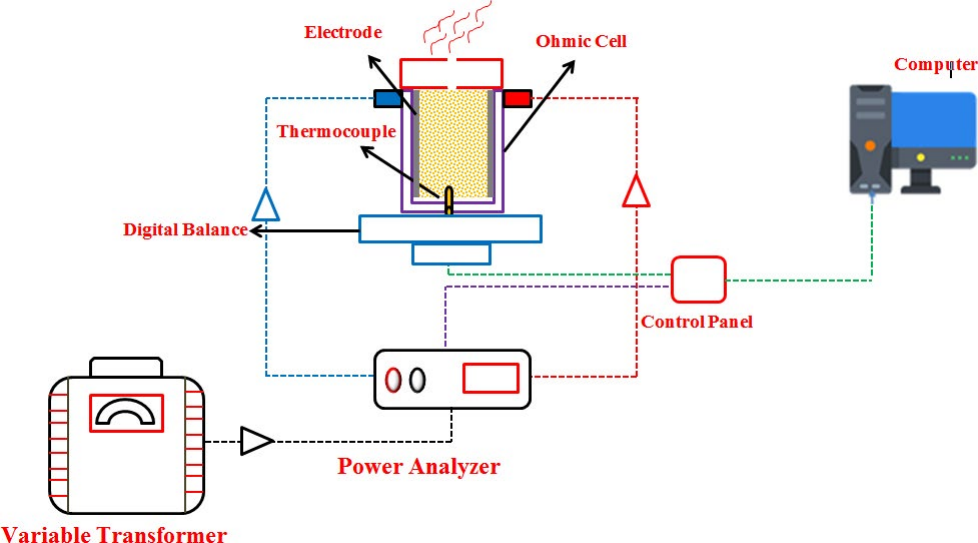
Schematic of the equipment used for the heating process of ohmic

A reservoir made of plastics thermoset was considered for this process, and the samples were poured into the reservoir between two electrodes and the initial temperature was recorded after stabilization and after recording the temperature, the voltage was applied to the set, and the samples were heated. Three heating gradients of 8.33, 10.83, and 13.33 V/cm were selected for the heating process. Approximately 10% (from 90 to 81 g), 20% (from 90 to 72 g), and 30% (from 90 to 63 g) of the total weight of the sour orange juice samples are poured into the steam cell and evaporated in the heating process. All the samples were 90 g. Figure 1 presents a schematic diagram of the heating process and the system components.

The experiments were conducted in a static Ohmic heating system. The system used consisted of a compact and transparent plastic cell (length 6 cm, width 6 cm, height 6 cm wall thickness of 0.3 cm), an electrode made of stainless steel (thickness of 0.1 cm) that the distance between the two electrodes is 6 cm, a variable transformer that is responsible for generating different voltages (3 kW, 0–300 V, 50 Hz, MST – 3, Toyo, Japan), a power analyzer (Lutron DW‐6090) responsible for monitoring the pattern of energy behavior of the system, a thermocouple, and a computer to store data with their profile. A scale (±0.01 g) was used to measure the cell weight and its contents during the process that was placed under the cell. All the experiments were conducted in the Biosystem Mechanics Department of Agricultural Sciences and Natural Resources of Gorgan University.

### 2.3 Energy analysis

Energy used in the drying and heating process is important for production processes in the industrial and household sectors. However, the price of energy is extremely expensive; therefore, there is a strong incentive to invent processes that will use energy efficiently. Currently, widely used drying and heating processes are complicated and inefficient; moreover, it is generally damaging to the environment. What is needed is a simplified, lower‐cost approach to this process one that will be replicable in a range of situations (Azadbakht, Vahedi Torshizi, Noshad, & Rokhbin, 2018).

**FIGURE 2:**
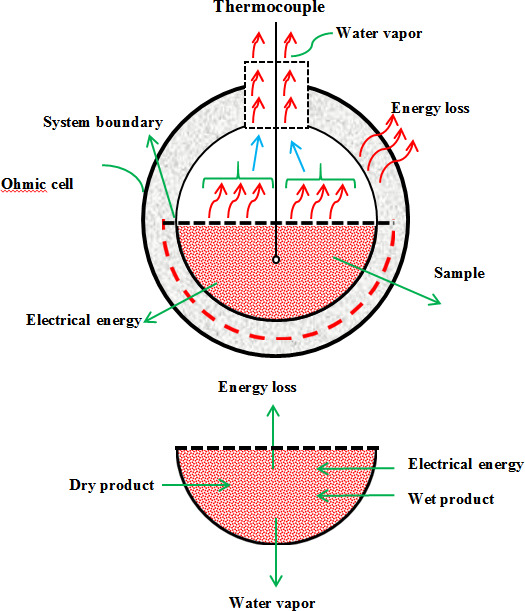
Volume control of the Ohmic heating system

Figure 2 shows the energy and mass conservation to control the volume of Ohmic heating (Ohmic cell). The general equation of mass conservation can be expressed in Equation (1):

(1) $$\sum_{m_{\mathrm{in}}=m_{\mathrm{ew}}+m_{P}}m_{\mathrm{in}}=\sum m_{\mathrm{out}}$$

The general equilibrium energy can be expressed by using Equation (2) that the input energy is equal to the output energy (Darvishi, Hosainpour, Nargesi, & Fadavi, 2015).

(2) $$\begin{array}{c} \sum E_{\mathrm{in}}=\sum E_{\mathrm{out}}\\ {\left(mC_{P}T\right)}_{\mathrm{in}}+E_{\mathrm{electrical}}={\left(mC_{P}T\right)}_{P}+m_{\mathrm{ew}}\lambda_{\mathrm{wp}}+E_{\mathrm{Loss}} \end{array}$$

The product heat (latent heat of the product) was calculated using Equation (3), and water heat (latent heat of water) was calculated using Equation (4) (Abdelmotaleb, El‐Kholy, Abou‐El‐Hana, & Younis, 2009; Sharqawy, Lienhard, & Zubair, 2010).

(3) $$\frac{\lambda_{\mathrm{wp}}}{\lambda_{w}}=1+23\times \exp\left(‐40m_{t}\right)M\geq 0.9\left(\frac{\text{kg Water}}{\text{Kg dry matter}}\right)$$

(4) $$\begin{array}{c} \lambda_{w}=3.217\times 10^{6}‐2.631\times 10^{3}T‐2.40T^{2}+1.460\times 10^{‐2}T^{3}‐2.079\times 10^{‐5}T^{4}\\ 273\leq T\leq 473K \end{array}$$

The heat capacity was calculated using the Siebel's model (Heldman & Moraru, 2014):

(5) $$C_{P}=0.71+3.393\times \frac{M_{t}}{1+M_{t}}1.86M_{t}\leq 19\left(\frac{\text{kg Water}}{\text{Kg dry matter}}\right),\;T>273K$$

The energy given to the system was recorded using the input current and the voltage given to the samples and calculated according to Equation (6) (Darvishi, Khostaghaza, & Najafi, 2013).

(6) $$E_{\text{electrical}}=\int_{0}^{t}\left(V\times I\right)dt$$

The system energy efficiency of Ohmic heating was calculated using Equation (8) (Darvishi et al., 2015).

(7) $$\eta_{\text{en}}=\frac{{\left(mC_{P}T\right)}_{P}+m_{\text{ew}}\lambda_{\text{wp}}}{{\left(mC_{P}T\right)}_{\text{in}}+E_{\text{electrical}}}$$

### 2.4 Exergy analysis

With the onset of the energy crisis, energy and exergy (the maximum useful work that comes from a certain amount of available energy or from the flow of materials) analyses are among the leading thermodynamic research works. In the exergy analysis, the main purpose is to determine the location and amount of irreversible production during the various processes of the thermodynamic cycle and the factors affecting the production of this irreversibility. In this way, in addition to evaluating the performance of various components of the thermodynamic cycle, methods to increase cycle efficiency are also identified (Azadbakht, Vahedi Torshizi, et al., 2018).

In the analytical range, the entrance (input) exergy, the output exergy and exergy loss of the ohmic heating system were investigated. Generally, the total exergy equation of the heating system is described using Equation (8).

(8) $$\begin{array}{c} \sum EX_{\mathrm{in}}=\sum EX_{\mathrm{out}}\\ m_{\mathrm{in}}ex_{\mathrm{in}}+E_{\mathrm{electrical}}=m_{P}ex_{P}+m_{\mathrm{ew}}ex_{\mathrm{ew}}+EX_{\mathrm{Loss}} \end{array}$$

The exergy loss was calculated using Equation (9) (Azadbakht, Aghili, Ziaratban, & Vehedi Torshizi, 2017).

(9) $$ex_{\mathrm{loss}}=EX_{\mathrm{in}}‐EX_{\mathrm{out}}$$

Exergy components were calculated at the input and output of the Ohmic heating system using Equation (10) (Azadbakht, Torshizi, Ziaratban, & Aghili, 2017).

(10) $$ex=C_{P}\left[\left(T‐T_{\infty }\right)‐T_{\infty }\ln\left(\frac{T}{T_{\infty }}\right)\right]$$

The exergy efficiency of the Ohmic system can be calculated using Equation (11) (Darvishi et al., 2015).

(11) $$\eta_{\mathrm{ex}}=\left(\frac{m_{P}ex_{P}+m_{\mathrm{ew}}ex_{\mathrm{ew}}}{m_{\mathrm{in}}ex_{\mathrm{in}}+E_{\mathrm{electrical}}}\right)\times 100$$

Van Gool (1997) suggests that the maximum improvement in the exergy efficiency of a process or system becomes apparent once the exergy loss or exergy irreversible becomes its lowest level. It was suggested that the concept of “improvement potential” would be widely used in analyzing the processes or different section of the economy, and Hammond and Stapleton presented this improvement potential in the evaluation form (Hammond & Stapleton, 2001).

(12) $$IP=\left(1‐\eta_{\mathrm{ex}}\right)\times \left(EX_{\mathrm{in}}‐EX_{\mathrm{out}}\right)$$

### 2.5 ANN modeling

**FIGURE 3:**
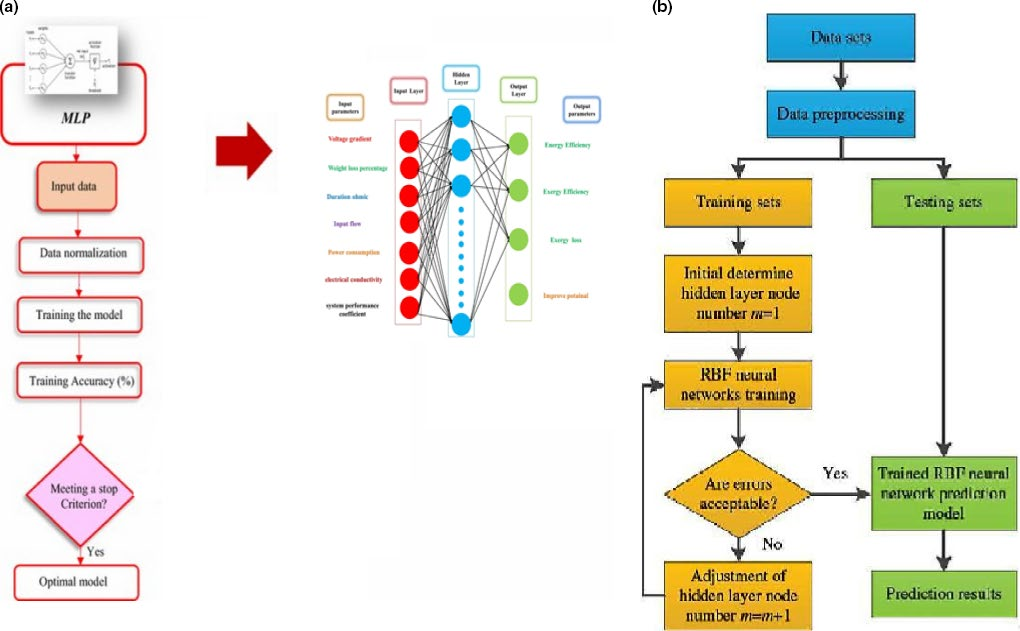
Neural Network Input and Output Schematic. A: Flowchart of the MLP B: Flowchart of the RBF (Bahiraei, Heshmatian, & Moayedi, 2019)

The training process is the important way to achieve a high accuracy from a developed model. The first step was to identify the dependent and independent variables. In this study, voltage gradient, weight loss percentage, during ohmic, input flow, power consumption, electrical conductivity and system performance coefficient (input variables) variables and energy efficiency, exergy efficiency, exergy loss and improve potential were considered as dependent variables (output variables). Therefore, by identifying the input and output variables, a MLP type of ANN was employed to develop the target model. As seen in Figure 3a, the architecture of these networks generally has three layers which include input, hidden, and output layers. Layers are connected by nodes; therefore each layer has its own node. The signal processing begins from first layer, also in Figure 3b showed RBF architecture. Receiving information from external nodes activates the related nodes on input layer and emits a signal to the next layer. Each connection between two nodes in two adjacent layers is related to each other by weighting coefficients, that this weights adjust the signal strength based on the input data. In training of the back propagation method, the error is determined by comparing the output and the desired output and this error is returned to the hidden and input layers of the next training processes. The network training operation ends when the error comes down below some value specified by the user (Najafi et al., 2018). In this research, the artificial multilayer perceptron (MLP) and radial basic function (RBF) neural network were used to model by Neuro Solution 5 software. Hyperbolic tangent, linear, and sigmoid activation functions (Equation 13 and 14), which are the most common type of activation functions, were used in the hidden input and output layer. In this paper, the Levenberg‐Marquardt algorithm was used to learn the network (Taheri‐Garavand, Karimi, Karimi, Lotfi, & Khoobbakht, 2018). Additionally, 70% of the data were used for training, 15% of them were used for network evaluation (Validating Data), and 15% of the data were used for testing the network (Testing data). Before training the model, the input‐output parameters in data sets were arranged, and the inputs in the data set were normalized between 0 and 1 range by using the Equation (20). Because of the output layer activation function is linear (purelin) in all architectures; only the input parameters were normalized by Equation (20). The voltage gradient, weight loss percentage, duration ohmic, Input flow, Power consumption, electrical conductivity and system performance coefficient as network inputs energy efficiency, exergy efficiency, exergy loss, and improve potential were the considered network outputs (Figure 3). A total of 5 Run were considered to achieve the minimum error rate and maximum network stability as a mean of 5,000 Epoch for the network. The error was estimated using an algorithm with back propagation error. Statistical parameters, including Root Mean Square Error (RMSE), *R* ^2^, and Mean Absolute Error (MAE) were calculated for inputs, and the relationships were calculated using the formulas shown in Table 1.

(20) $$I_{\text{norm}}=\frac{I‐I_{\min}}{I_{\max}‐I_{\min}}$$

**TABLE 1:** Neural network equations

Formula	Formula number	Reference
$\text{Tanh}\;=\frac{e^{x}‐e^{‐x}}{e^{x}+e^{‐x}}$	(13)	Azadbakht, Vehedi Torshizi, Aghili, and Ziaratban (2018)
$\text{Sig}=\frac{1}{1+e^{‐x}}$	(14)	Salehi, Gohari, Nemati, and Latifi Darab (2017)
$R^{2}=1‐\frac{\sum_{i=1}^{n}{\left(P_{i}‐O_{i}\right)}^{2}}{{\left(P_{i}‐O\right)}^{2}}$	(15)	Azadbakht, Aghili, Ziaratban, and Torshizi (2017)
$R=\sqrt{1‐\frac{\sum_{i=1}^{n}{\left(P_{i}‐O_{i}\right)}^{2}}{{\left(P_{i}‐O\right)}^{2}}}$	(16)	Khoshnevisan, Rafiee, and Omid (2013)
$\text{MSE}=\sum_{i=1}^{n}\frac{{\left(P_{i}‐O_{i}\right)}^{2}}{n}$	(17)	Azadbakht, Torshizi, and Ziaratban (2016)
$\text{RMSE}=\sqrt{\sum_{i=1}^{n}\frac{{\left(P_{i}‐O_{i}\right)}^{2}}{n}}$	(18)	Khoshnevisan et al. (2013)
$\text{MAE}\;=\frac{\sum_{i=1}^{n}\left|P_{i}‐O_{i}\right|}{n}$	(19)	Azadbakht et al. (2016)

Note

The Equations (15–19) include the predicted values (*P_i_*) and the actual values (*O_i_*) and the mean value of the data (*O*).

In Equation (20), *I*_norm_ is the normalized data, *I* is the measured data, *I*_min_ is the least measured, and *I*_max_ is the most measured data.

### 2.6 Analyze the response surface method

The response surface method (RSM) is a series of statistical and mathematical approaches used to analyze experimental results (Han, Li, Wu, & Shao, 2015). This method is also very useful in designing, improving, and formulating new products. The grade 2 model is suitable for industrial processes and has much strength. Also, using the ANOVA analysis, the models presented for the responses were evaluated and regression coefficients were estimated for linear, interactions and grade 2 sentences and the fitting quality of the models equation was expressed using the convergence coefficient (*R* ^2^) (Myers, Montgomery, & Anderson‐Cook, 2009; Khuri, 2006). In order to investigate the properties and optimization of system performance factor, heating process duration, input current, power consumption and the electrical conductivity of sour orange juice during the heating process, the surface response method, a central composite design (CCD) with 5 central points and with Design Expert 11 software was used (Table 2).

**TABLE 2:** Optimization values for artificial neural network parameters for MLP and RBF networks

Number of hidden layers	Learning rule	Type of activation function	The number of hidden layer neurons	Testing data (%)	Validating data (%)	Training data (%)
1	Levenberg Marquardt	Hyperbolic tangent	4	15	15	70
1	Levenberg Marquardt	Sigmoid	4	15	15	70
1	Levenberg Marquardt	Liner	4	15	15	70

In order to investigate the properties and optimization of energy and exergy of sour orange juice during the heating process, the surface response method, a central composite design (CCD) with 5 central points and with Design Expert 11 software was used. In this study, the independent variables were voltage gradients and percentage of weight loss (Table 1), dependent variables were energy efficiency, exergy efficiency, exergy loss, and improvement potential as responses to investigate the process of the desired changes to the levels of independent variables. Finally, the goal is to examine the values predicted by the response surface methodology.

## 3 RESULTS AND DISCUSSION

Table 3 presents the results of networks with different activation functions and different RBF and MLP networks. According to obtained results for the energy efficiency, the best value of *R* ^2^ and the lowest value of *MSE* were 0.96999 and 0.9494 in a network with the linear activation function and the MLP network, respectively. The best value of *R* ^2^ for exergy efficiency and the lowest value of *MSE* in a RBF network with the hyperbolic tangent activation function were 0.9994 and 0.165, respectively. For the improvement potential, the best value of *R* ^2^ and the lowest value of *MSE* were 0.996 and 0.0088 in the MLP network and the linear activation function, respectively. Furthermore, for the exergy loss, the best value of *R* ^2^ and the lowest value of *MSE* were 0.99650 and 0.01379, respectively, in the RBF network with a linear activation function. Given the high values of *R* ^2^ for all networks as well as low levels of *MSE* for networks and activation functions, all networks have the proper ability to predict and train, but the best value of *R* ^2^ and the lowest value of *MSE* in the above‐reported networks. Table 4 presents the rest of details of all networks. For the energy efficiency, exergy, and improvement potential, the best value of *R* ^2^ and the lowest value of *MSE* were seen in the MLP network; and this network had better values than the RBF network. For the exergy loss, the RBF network could get better values. Among three activation functions, the best values of *R* ^2^ and *MSE* were obtained for the linear activation function for energy efficiency, improvement potential, and exergy loss; and the best values were shown for the energy efficiency of the hyperbolic tangent activation function.

**TABLE 3:** Error values in predicting experimental data using optimal artificial neural network

		*MSE*	NMSE	MAE	Min‐AE	Max‐AE	*R* ^2^
*Energy efficiency*							
Linear	MLP	0.9494	0.0069	0.7881	0.0178	2.23	0.9969
	RBF	4.50785	0.02956	1.0149	0.00603	5.62957	0.99124
Sigmoid	MLP	1.1626	0.0092	0.863	0.0434	2.2258	0.9959
	RBF	9.67505	0.05418	1.41004	0.0005	8.65822	0.98255
Tangent hyperbolic	MLP	1.50066	0.01142	1.02068	0.03776	2.39236	0.99465
	RBF	10.68	0.089	1.0365	0.000569	11.398	0.9812
*Exergy efficiency*							
Linear	MLP	0.8948	0.0093	0.6454	0.0485	2.3638	0.9981
	RBF	4.34238	0.04863	0.87211	0.00164	7.38578	0.97937
Sigmoid	MLP	1.4898	0.0153	1.0126	0.0351	2.5973	0.9928
	RBF	0.22013	0.00266	0.27113	0.00199	1.57278	0.99935
Tangent hyperbolic	MLP	1.08538	0.01821	0.88927	0.08002	2.40489	0.99334
	RBF	0.165	0.0021	0.126	0.0021	1.25	0.9994
*IP*							
Linear	MLP	0.0088	0.0086	0.0715	0.0014	0.235	0.996
	RBF	0.03384	0.02625	0.10156	0.00003	0.55283	0.99388
Sigmoid	MLP	0.0362	0.0335	0.1482	0.0081	0.3759	0.9846
	RBF	0.08072	0.05145	0.13232	0.00041	0.90488	0.98418
Tangent hyperbolic	MLP	0.08858	0.07648	0.23443	0.0043	0.76062	0.96419
	RBF	0.0958	0.0659	0.165	0.00005	1.005	0.9713
*Exergy loss*							
Linear	MLP	0.0255	0.0151	0.1176	0.0027	0.4701	0.9925
	RBF	0.01379	0.007	0.0562	0.00144	0.41489	0.9965
Sigmoid	MLP	0.0397	0.0341	0.1641	0.0072	0.3584	0.9831
	RBF	0.01805	0.00907	0.0672	0.00019	0.44747	0.99546
Tangent hyperbolic	MLP	0.04323	0.02978	0.16156	0.00165	0.54642	0.9863
	RBF	0.0195	0.0123	0.0789	0.00021	0.489	0.992

**TABLE 4:** Independent variables of test surface

Variable	Level	
	−1	+1
Voltage gradient (V/cm)	8.33 (50 v)	13.33 (80 v)
Percentage weight loss (%)	10	30

**FIGURE 4:**
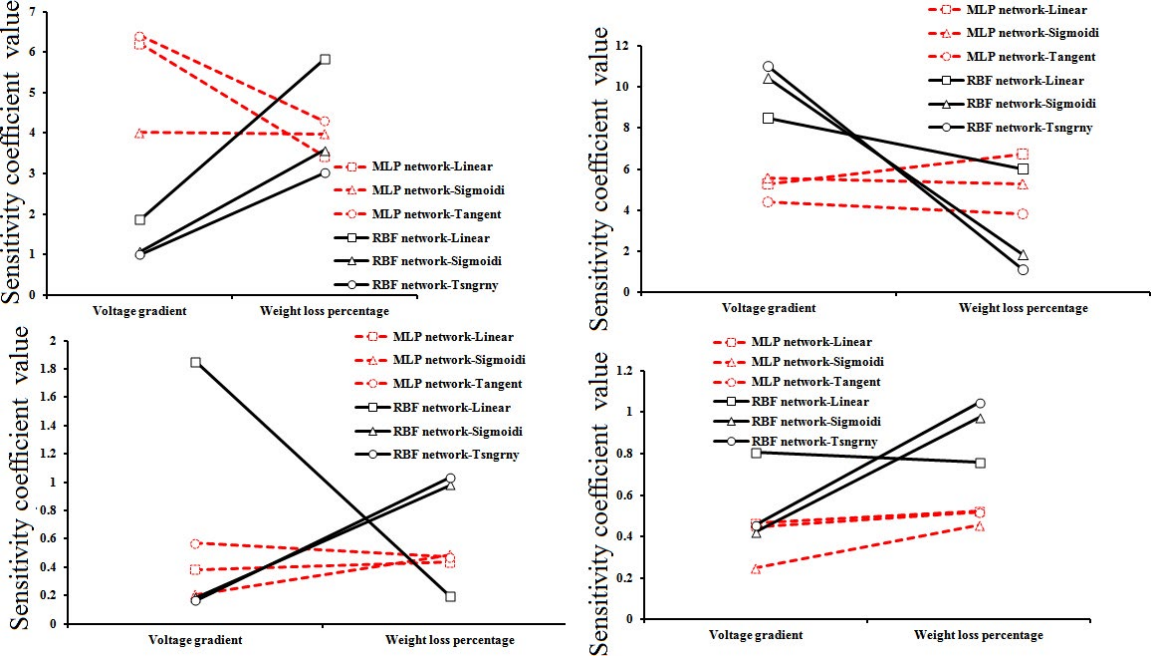
Sensitivity coefficient energy efficiency, exergy efficiency, improve potential, and exergy loss for sour orange juice during the ohmic heationg process

According to obtained results from the data sensitivity coefficient (Figure 4), the higher sensitivity to weight loss belonged to the energy efficiency of RBF network, and also the higher sensitivity to voltage in the MLP network. The obtained coefficients in this network were similar to obtained coefficients in the response surface method. However, the sensitivity of two networks was higher for the weight loss percentage. It can be argued that the weight loss percentage had a higher sensitivity to energy efficiency of the ohmic process than the process voltage. For exergy efficiency, the higher sensitivity was obtained for voltage gradient than weight loss percentage of the RBF network. In general, the voltage gradient had a higher sensitivity to the weight loss percentage, and it was similar to obtained coefficients for weight loss percentage and voltage gradient in the response surface method. For the improvement potential and the exergy loss, the obtained sensitivity coefficients by both networks for weight loss were higher than the voltage gradient. The results were similar to the response surface method. In this method, the weight loss had a greater effect (Table 5).

**TABLE 5:** Results of the *R*‐level response method

	Energy efficiency (%)	Exergy efficiency (%)	Exergy loss (KW)	Improvement potential (KW)
*R* ^2^	0.9932	0.95	0.99	0.97
*R* ^2^ adjusted	0.9909	0.91	0.98	0.96
*R* ^2^ predicted	0.9845	0.63	0.94	0.94
Adeq precision	69.90	17.10	46.61	40.73

The results of the Sequential Model Sum of Squares show that how complex phrase participates in the final model. Table 6 shows the results of the models for the energy efficiency, the exergy efficiency, the improvement potential, and the exergy loss. The linear and factors interaction model was selected as the best model for the energy efficiency, and the second‐order model versus the two factors was chosen as the best model for the exergy efficiency. The best models for the improvement potential and for the exergy loss were proposed the average and linear model and the second‐order model versus the two factors, respectively.

**TABLE 6:** Best models formed for data

Source	Sum of squares	*df*	Mean square	*F*‐value	*p*‐value	
*Energy efficiency* (%)						
Mean versus Total	67,598.65	1	67,598.65			
Linear versus Mean	1,411.51	2	705.75	334.42	<.0001	
2FI versus Linear	11.32	1	11.32	10.42	.0104	Suggested
Quadratic versus 2FI	4.88	2	2.44	3.49	.0890	
Cubic versus Quadratic	1.28	2	0.6407	0.88	.4686	Aliased
Residual	3.62	5	0.7237			
Total	69,031.26	13	5,310.10			
*Exergy efficiency* (%)						
Mean versus Total	24,294.40	1	24,294.40			
Linear versus Mean	788.10	2	394.05	28.25	<.0001	
2FI versus Linear	9.81	1	9.81	0.6811	.4306	
Quadratic versus 2FI	84.32	2	42.16	6.51	.0253	Suggested
Cubic versus Quadratic	43.58	2	21.79	61.69	.0003	Aliased
Residual	1.77	5	0.3532			
Total	25,221.98	13	1940.15			
*Improvement potential* (KW)						
Mean versus Total	180.75	1	180.75			
Linear versus Mean	11.59	2	5.80	167.42	<.0001	Suggested
2FI versus Linear	0.0016	1	0.001	0.0407	.8445	
Quadratic versus 2FI	0.087	2	0.04	1.18	.3613	
Cubic versus Quadratic	0.17	2	0.08	5.52	.0543	Aliased
Residual	0.0804	5	0.0161			
Total	192.69	13	14.82			
*Exergy loss* (KW)						
Mean versus Total	196.14	1	196.14			
Linear versus Mean	12.41	2	6.21	76.98	<.0001	
2FI versus Linear	0.5883	1	0.5883	24.31	.0008	
Quadratic versus 2FI	0.1185	2	0.0592	4.18	.0640	Suggested
Cubic versus Quadratic	0.0902	2	0.0451	24.81	.0025	Aliased
Residual	0.0091	5	0.0018			
Total	209.36	13	16.10			

**FIGURE 5:**
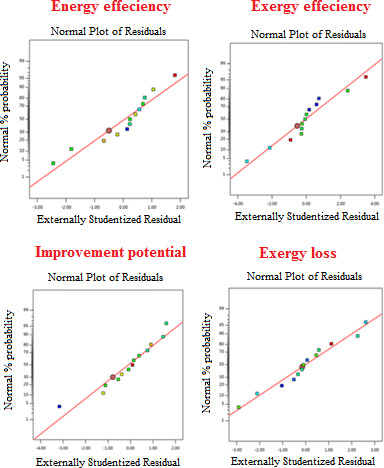
Measured Scattering values for response obtained by the response surface method

Table 7 shows the results of runs and Epoch for the network creation. This table generally considers the energy efficiency, exergy efficiency, exergy, and exergy loss, and improvement potential because network output layers are composed of these factors. According to obtained results, the MLP network with a linear activation function had the best value and the fastest time for network training until the creation because it had the lowest amount of running and repetition; and the network was created after 541 Epoch per run that was better than other created networks. According to the above table, which presents the best values of topology, the best values of *R* ^2^ and *MSE* are seen in the same network that is certainly the best network. In addition, also in Figure 5 is showed the dispersion value of errors for RSM. Given that the values obtained are very close to the line drawn, it can be stated that the dispersion values of the error obtained are appropriate for the energy efficiency, the exergy efficiency, the improvement potential, and the exergy loss. This indicates that the difference between actual values and predicted values by the model is appropriate, so that it has been able to obtain values close to the line creating an appropriate normal distribution. The results show the compatibility of the model and actual data with the predicted data.

**TABLE 7:** Some RBF and MLP neural network topologies to predict the values of training and cross‐validation for energy efficiency, Exergy efficiency, exergy, and the potential for improvement of sour orange juice during the ohmic heating process

Activation function	Network	Train		Cross‐validation	
		Run	Epoch	Run	Epoch
Linear	MLP	1	541	3	32
	RBF	2	594	3	52
Sigmoid	MLP	2	236	5	16
	RBF	3	4,569	1	68
Tangent hyperbolic	MLP	1	3,200	3	100
	RBF	3	895	4	256

Table 8 presents the results of the prediction by the neural network and the response surface method as actual values. The standard deviation was obtained from results of the neural network and the response surface method as presented in Table 9. Given that the lower standard deviation indicates the proximity of predicted number to the real number, it can be concluded that the neural network with RBF has the least standard deviation compared with the created network by MLP and the response surface method for energy efficiency, exergy efficiency, exergy loss, and improvement potential. For energy efficiency and exergy loss, predicted values with the MLP neural network and the response surface method were almost similar, and it can be argued that they had the same predictive power, but the efficacy of exergy and the improvement potential of MLP neural network got better values for the prediction. In general, it can be concluded that the linear and sigmoid activation functions for both networks were able to obtain best values for the hyperbolic tangent network.

**TABLE 8:** The predicted value by the inductive neural network and the response surface method

Response	Observed	Predicted RSM	4‐sig‐MLP	4‐lin‐MLP	4‐tan‐MLP	4‐sig‐RBF	4‐lin‐RBF	4‐tan‐RBF
Energy efficiency	72.36	72.1103	71.54779	71.41792	71.80781	72.3605	70.41656	72.37721
	70.489	72.1103	71.35711	71.19299	71.92676	71.89976	70.35051	72.49616
	64.56	64.3303	64.83116	64.85904	64.03192	64.51593	65.29973	64.60132
	79.12	79.664	77.15137	79.00387	82.09885	79.10655	79.65952	82.66825
	70.145	73.6663	71.69	71.19299	72.04618	71.44216	70.28126	72.61558
	69.6	69.1411	69.00717	67.7227	68.71553	69.4531	69.6061	69.28493
	91.68	90.6395	89.45423	92.68	91.45612	91.28867	92.85756	92.02552
	60.21	59.6786	59.63436	59.79872	58.49423	60.40025	60.78136	59.06363
	83.91	84.542	83.30342	83.48424	83.44715	83.27504	84.04281	84.01655
	65.05	64.3303	64.83116	64.6274	63.23292	65.0986	65.10975	63.80232
	50.31	50.2161	52.91763	51.51618	52.10174	58.16036	55.93957	52.67114
	79.69	79.8903	80.69279	80.56565	82.56887	78.8672	79.74082	83.13827
	80.31	79.8903	81.6538	81.35998	82.1105	80.33035	80.20512	82.6799
Exergy efficiency	43	43.6266	44.03704	43.25637	43.2318	43.00199	43.67023	43.8012
	43.65	43.6266	43.81679	43.4015	43.12032	42.90811	43.58317	43.68972
	47.4	47.9575	47.35613	47.51712	48.13013	47.42146	46.8221	48.69953
	59.146	59.7478	59.40479	59.25656	56.65161	60.16229	59.65859	57.22101
	43.45	41.8822	43.89	43.4015	43.00919	42.81532	43.51655	43.57859
	31.569	30.763	30.40603	29.99622	32.09437	31.3115	36.68511	32.66377
	38.45	41.2295	39.00566	39.09086	38.18184	38.52267	38.77311	38.75124
	37.65	40.8457	35.05273	37.32887	35.24511	37.5849	37.27982	35.81451
	58.42	54.4447	57.04668	58.6356	57.06416	58.37279	58.28597	57.63356
	47.321	47.9575	47.35613	47.45162	48.04213	47.29561	47.32456	48.61153
	46	43.6103	47.74237	46.53383	45.27384	45.83913	45.683	45.84324
	32.36	31.9777	33.93066	29.99622	33.95386	31.96892	33.11944	34.52326
	33.569	31.9777	34.868	29.99622	33.82581	33.5379	33.06595	34.39521
IP	3.8	3.72877	3.756508	3.784321	3.633192	3.798337	3.666752	4.202592
	3.756	3.72877	3.770001	3.809866	3.607458	3.837811	3.709424	4.176858
	4.568	4.63652	4.413093	4.672968	4.258543	4.570037	4.206775	4.827943
	3.561	3.54214	3.922476	3.586717	2.930947	3.561773	3.574494	3.500347
	3.712	3.54722	3.98	3.809866	3.581729	3.876496	3.760671	4.151129
	3.65	3.73385	3.664985	3.694098	4.410618	3.654355	3.649102	4.980018
	4.45	4.6416	4.636405	4.570662	4.744511	4.484254	4.388045	5.313911
	4.8	4.97406	4.837882	4.842654	5.034232	4.790282	4.868057	5.603632
	3.056	2.81594	3.076267	3.07958	3.130366	3.066333	3.005007	3.699766
	4.789	4.63652	4.413093	4.688111	4.288546	4.788594	4.671496	4.857946
	5.563	5.54935	5.320204	5.56982	5.453985	4.887987	6.049833	6.023385
	2.95	2.82102	2.912029	2.948563	3.311391	3.18312	3.024273	3.880791
	3.05	2.82102	2.803515	2.836571	3.324186	3.048867	3.003506	3.893586
Exergy loss	3.894	3.83163	3.606282	3.800976	3.710741	3.895917	3.79466	4.280141
	3.795	3.83163	3.613643	3.81672	3.711049	3.848074	3.800537	4.280449
	4.698	4.70803	4.696401	4.786734	4.699647	4.704625	4.77164	5.269047
	3.456	3.47036	3.814438	3.372919	3.558676	3.456453	3.444699	4.128076
	3.814	3.64288	3.79	3.81672	3.711447	3.800447	3.809647	4.280847
	3.71	3.60197	3.521453	3.643101	3.807768	3.71839	3.711848	4.377168
	2.4	2.46197	2.492015	2.301818	2.151869	2.381031	2.356104	2.721269
	4.8	4.97406	4.837882	4.842654	5.034232	4.790282	4.868057	5.603632
	3.233	3.06706	3.138474	3.315022	3.125322	3.306656	3.24435	3.694722
	4.756	4.70803	4.696401	4.835374	4.748279	4.748452	4.725395	5.317679
	6.3	6.23397	6.51404	6.619889	6.5661	6.613811	6.521788	7.1355
	2.85	2.84303	2.772129	2.713094	2.641756	2.843157	2.848555	3.211156
	2.79	2.84303	2.756843	2.671262	2.565498	2.788634	2.801703	3.134898

**TABLE 9:** Standard deviation values of values predicted by neural network and response surface method

Response	Predicted RSM	4‐sig‐MLP	4‐lin‐MLP	4‐tan‐MLP	4‐sig‐RBF	4‐lin‐RBF	4‐tan‐RBF
Energy efficiency	0.176565	0.57432	0.666152	0.390459	0.000355	1.374217	0.012167
	1.146432	0.613846	0.497793	1.016651	0.997557	0.097928	1.419277
	0.162422	0.191737	0.21145	0.373409	0.031164	0.523067	0.029217
	0.384666	1.392031	0.082114	2.106369	0.009514	0.381495	2.508995
	2.489935	1.09248	0.741038	1.344338	0.917229	0.096347	1.746965
	0.324491	0.419198	1.327452	0.625413	0.103875	0.004314	0.222787
	0.735745	1.57386	0.707107	0.158308	0.276713	0.832659	0.244318
	0.375757	0.407039	0.290816	1.213232	0.134531	0.404014	0.810605
	0.446891	0.428919	0.301061	0.327283	0.448988	0.093908	0.075344
	0.508905	0.154745	0.298822	1.284871	0.034369	0.042248	0.882244
	0.066397	1.843873	0.8529	1.266951	5.551042	3.980706	1.669577
	0.141633	0.70908	0.619175	2.035667	0.581809	0.035936	2.438293
	0.296773	0.950213	0.742448	1.273146	0.014392	0.07416	1.675773
Exergy efficiency	0.443073	0.733296	0.181278	0.163907	0.001409	0.473926	0.566534
	0.016546	0.117941	0.175716	0.374543	0.524597	0.047254	0.028084
	0.394212	0.03102	0.082816	0.516283	0.015175	0.408638	0.91891
	0.425537	0.18299	0.078177	1.763803	0.718626	0.362456	1.361176
	1.108602	0.311127	0.034295	0.311697	0.448788	0.047055	0.09093
	0.569928	0.822342	1.112122	0.371495	0.182082	3.617636	0.774122
	1.965403	0.392914	0.453159	0.189618	0.051385	0.228476	0.213009
	2.259701	1.836549	0.22707	1.700511	0.04603	0.261758	1.297885
	2.810962	0.971084	0.15245	0.958724	0.033381	0.094776	0.556097
	0.450073	0.024842	0.092361	0.509916	0.017955	0.002514	0.912542
	1.689773	1.23204	0.377476	0.513473	0.113754	0.224152	0.110847
	0.270327	1.110625	1.671443	1.127032	0.276538	0.537003	1.529658
	1.125219	0.918533	2.526335	0.181591	0.021992	0.355708	0.584218
IP	0.050367	0.030754	0.011087	0.117951	0.001176	0.094221	0.284676
	0.019255	0.0099	0.038089	0.105035	0.057849	0.032934	0.297591
	0.048451	0.109536	0.074224	0.218819	0.00144	0.255424	0.183807
	0.013336	0.255602	0.018185	0.445515	0.000547	0.009542	0.042888
	0.116517	0.189505	0.069201	0.092115	0.116316	0.034415	0.310511
	0.059291	0.010596	0.031182	0.537838	0.003079	0.000635	0.940465
	0.239844	0.209407	0.131961	0.178149	0.117701	0.009349	0.580776
	0.135482	0.131808	0.085321	0.208251	0.024221	0.043809	0.610877
	0.123079	0.026786	0.030161	0.165627	0.006872	0.048123	0.568253
	0.169748	0.014331	0.016674	0.052585	0.007307	0.036057	0.455211
	0.10782	0.265806	0.071339	0.353875	0.000287	0.083088	0.048752
	0.009652	0.171683	0.004823	0.077085	0.477306	0.344243	0.325541
	0.091203	0.026849	0.001016	0.255542	0.164841	0.052519	0.658169
Exergy loss	0.161913	0.174291	0.150917	0.193879	0.000801	0.032876	0.596506
	0.044102	0.203448	0.065778	0.129584	0.001356	0.070244	0.273043
	0.025901	0.128239	0.015358	0.059363	0.037529	0.003915	0.343264
	0.007092	0.001131	0.062744	0.001164	0.004684	0.052071	0.403791
	0.010154	0.253454	0.058747	0.072603	0.00032	0.007991	0.475229
	0.121	0.016971	0.001923	0.072516	0.009583	0.003078	0.330111
	0.076389	0.133323	0.047305	0.069133	0.005933	0.001306	0.471759
	0.043819	0.065064	0.069425	0.175455	0.013413	0.031039	0.227172
	0.123079	0.026786	0.030161	0.165627	0.006872	0.048123	0.568253
	0.117337	0.06684	0.057998	0.07614	0.052082	0.008026	0.326487
	0.03392	0.042143	0.056126	0.005459	0.005337	0.021641	0.397167
	0.04669	0.151349	0.226196	0.188161	0.221898	0.156827	0.590788
	0.004929	0.055063	0.096807	0.147251	0.004839	0.001022	0.255376

## 4 CONCLUSION

According to results of the generated neural network for energy efficiency, exergy efficiency, exergy loss and improvement potential, rate of predicted *R* ^2^ was very acceptable; and the network‐predicted data were close to real data. The MLP network was better than RBF because its *R* ^2^ and *MSE* values were lower than the RBF network. The linear activation function was the best choice for activation. It should be noted that the hyperbolic tangent activation function had the best values of *R* ^2^ and *MSE* for the exergy efficiency. In terms of network training, MLP network with linear activation function could have the fastest training because it was able to create a very good network with the least repetition and execution. According to predicted values by the artificial neural network and the response surface method, the accuracy of neural network was higher than the response surface method to predict values.

## Nomenclature

*C*_p_: heat capacity (J/kg K)
*E*_electrical_: electricity energy (J)
*E*: energy (J)
*E_loss_*: energy loss (J)
*E*_sc_: specific energy consumption (J/kg water)
*EX*: exergy (J)
*Ex*: specific exergy (J/kg water)
*EX_loss_*: exergy loss (J)
*I*: current intensity (A)
IP: improvement potential (J)
*M*: mass (kg)
*M*: moisture content (kg water/kg dry matter)
*M_t_*: moisture content at any time (kg water/kg dry matter)
*R*^2^: coefficient of determination (−)
*T*: temperature (K)
*T*: time (s)
*T*_ref_: ambient temperature (K)
*V*: voltage (V)
*Η*_en_: energy efficiency (%)
*Η*_ex_: exergy efficiency (%)
Λ*w*: latent heat of pure water (J/kg)
Λ*w*_p_: latent heat of sample (J/kg)

**Subscripts**
0: initial
In: inlet
Out: outlet
*P*: product
*E*_w_: water evaporated
∞: ambient
*C*: Ohmic cell
*E*: electrode
